# Analysis and prediction of liver volume change maps derived from computational tomography scans acquired pre- and post-radiation therapy

**DOI:** 10.1088/1361-6560/acfa5f

**Published:** 2023-10-04

**Authors:** Guillaume Cazoulat, Aashish C Gupta, Mais M Al Taie, Eugene J Koay, Kristy K Brock

**Affiliations:** 1 Department of Imaging Physics, The University of Texas MD Anderson Center, Houston, TX, United States of America; 2 Department of Radiation Oncology, The University of Texas MD Anderson Center, Houston, TX, United States of America

**Keywords:** liver cancer, radiation therapy, deformable image registration, deep learning, treatment response

## Abstract

External beam radiation therapy (EBRT) of liver cancers can cause local liver atrophy as a result of tissue damage or hypertrophy as a result of liver regeneration. Predicting those volumetric changes would enable new strategies for liver function preservation during treatment planning. However, understanding of the spatial dose/volume relationship is still limited. This study leverages the use of deep learning-based segmentation and biomechanical deformable image registration (DIR) to analyze and predict this relationship. Pre- and Post-EBRT imaging data were collected for 100 patients treated for hepatocellular carcinomas, cholangiocarcinoma or CRC with intensity-modulated radiotherapy (IMRT) with prescription doses ranging from 50 to 100 Gy delivered in 10–28 fractions. For each patient, DIR between the portal and venous (PV) phase of a diagnostic computed tomography (CT) scan acquired before radiation therapy (RT) planning, and a PV phase of a diagnostic CT scan acquired after the end of RT (on average 147 ± 36 d) was performed to calculate Jacobian maps representing volume changes in the liver. These volume change maps were used: (i): to analyze the dose/volume relationship in the whole liver and individual Couinaud’s segments; and (ii): to investigate the use of deep-learning to predict a Jacobian map solely based on the pre-RT diagnostic CT and planned dose distribution. Moderate correlations between mean equivalent dose in 2 Gy fractions (EQD2) and volume change was observed for all liver sub-regions analyzed individually with Pearson correlation *r* ranging from −0.36 to −067. The predicted volume change maps showed a significantly stronger voxel-wise correlation with the DIR-based volume change maps than when considering the original EQD2 distribution (0.63 ± 0.24 versus 0.55 ± 23, respectively), demonstrating the ability of the proposed approach to establish complex relationships between planned dose and liver volume response months after treatment, which represents a promising prediction tool for the development of future adaptive and personalized liver radiation therapy strategies.

## Introduction

Primary liver cancers, including hepatocellular carcinomas (HCC) and intra-hepatic bile duct cancer (cholangiocarcinoma, CC), as well as secondary liver cancers from colorectal metastasis (CRC) are a leading cause of mortality worldwide (Ananthakrishnan *et al* 2006, Sung *et al* 2021). Among various treatment strategies for those cancers, external beam radiation therapy (EBRT) is a common option for unresectable tumors. A challenge associated with the treatment plan optimization of EBRT is the risk of radiation-induced liver disease (RILD), liver cells being particularly sensitive to radiation (Kim and Jung 2017). This risk of dose-dependent toxicity has been considerably lowered in the past few years with the advent of intensity-modulated radiotherapy (IMRT) and daily image guidance techniques, whose combination enabled the increase in dose to the tumor and ability to spare the remainder of the normal liver. While the incidence of RILD has decreased (Pan *et al* 2010), liver atrophy as a result of tissue damage and hypertrophy as a result of liver regeneration can still be observed weeks or months following the end of radiotherapy (Olsen *et al* 2009, Imada *et al* 2010, Su *et al* 2021). Predicting spatially the ability of the liver to regenerate after RT would enable further optimization of the treatment plan to preserve liver function. However, understanding of the spatial dose/volume relationship is still limited and the clinical question remains: is it better to optimize the plan to limit the high dose to normal liver tissue at the expense of a low dose bath to a larger volume of the liver (typically observed in volumetric modulated arc therapy) or to limit the volume of normal liver tissue receiving any dose at the expense of a limited about of normal liver tissue receiving a high radiation dose (typically observed in IMRT with a small number of treatment beams). Current dose constraints for liver are mostly based on mean dose to the normal liver known to be associated with the risk of RILD (Dawson *et al* 2002, Dawson and Ten Haken 2005, Pan *et al* 2010). In a study of 203 patients treated with focal liver irradiation, Dawson *et al* reported median values of mean liver doses of 31.3 and 37 Gy in patients who did not and did develop RILD, respectively (Dawson *et al* 2002). In addition to sparing the normal liver, current conformal techniques will aim at avoiding irradiation of the central hepatobiliary tract, responsible for other forms of liver toxicities (Toesca *et al* 2017). While these dose indications allowed to minimize the risk of liver toxicities, there is to date no clear understanding of the relationship between the resulting complex spatial distribution of the dose and events of liver atrophy or hypertrophy after treatment.

The analysis of those radiation-induced volume changes between longitudinal CT scans would require a consistent definition of sub-structures, which could be achieved for example by delineating the Couinaud’s liver segments, a classification used for liver surgery planning (Couinaud 1957). However, this task is time-consuming and potentially prone to observer variability. Deformable image registration (DIR) between the longitudinal images allows in theory to generate maps of expanding and contracting tissues by computing the determinant of the Jacobian matrix of the displacement vector field (DVF). This approach presents the advantage of providing estimates of the volume change at the voxel-level which would facilitate the correlation with dose distributions or functional imaging of the liver. However, DIR between pre- and post-RT CT scans is challenging (Sen *et al* 2020) and has been addressed by very few studies (Fukumitsu *et al* 2017, Cazoulat *et al* 2019, Anderson *et al* 2021).

Tumor regression or disease progression, as well as potential modification of image acquisition protocol, often create inconsistencies in image intensity information, causing traditional intensity-based DIR methods to estimate erroneous anatomical changes. The use of DIR methods driven by the liver contours only are particularly interesting in cases of mechanical-only induced liver deformations (Polan *et al* 2017, Sen *et al* 2020), but they do not have the ability to estimate internal complex volume changes that could occur as a response to radiation. To address this, a biomechanical-based DIR method driven only by liver and vasculature has been proposed (Cazoulat *et al* 2019). The reported accuracy and robustness of this method in aligning vessel bifurcation landmarks between pre- and post-RT cholangiocarcinoma CT scans suggests that it could also provide reliable volume change maps, reflecting radiation-induced changes, through the calculation of the Jacobian determinant.

In this paper, we leverage this previously validated DIR method to generate Jacobian maps describing liver volume change between pre- and post-RT liver diagnostic CTs for a large cohort of 100 patients. The goals of this study were (i): to analyze the relationship between these automatically generated Jacobian maps and planned dose distributions in the whole liver and segments; and (ii): to investigate the use of deep-learning to predict a Jacobian map solely based on the pre-RT diagnostic CT and planned dose distribution.

## Materials and methods

### Patient data

Imaging data and radiation therapy plans from 100 patients treated for primary or secondary liver cancer with radiation therapy at MD Anderson Cancer Center (Houston, TX) between 2015 and 2022 were retrospectively analyzed under an IRB approved protocol. Patients were selected based on availability of the data required for this study. All patients were treated for HCC, CC or CRC with IMRT and prescription doses ranging from 50 to 100 Gy delivered in 10–28 fractions. Patient characteristics are summarized in table 1. For each patient the following was collected: their planning CT scan with planned dose distribution, the portal and venous (PV) phase of a diagnostic CT scan acquired before RT planning (on average 15 ± 14 d), and a PV phase of a diagnostic CT scan acquired after the end of RT (on average 147 ± 36 d, range: 79–257 d). For 5 patients for whom no pre-RT diagnostic CTs were available, their planning CT was used in place. All diagnostic CTs had a slice spacing of 2.5 or 3 mm and fully encompassed the liver.

**Table 1.** Patient characteristics.

Gender: Male/Female	58/42
Cancer type: HCC, CC, Mets	25/64/11
Prescription: 50/52/52.5/60/62.5/65/67.5/69/70/75/100	6/1/3/33/1/2/31/1/9/10/3
Number of fractions: 10/13/15/23/25/28	12/1/67/1/18/1

All images and treatment plans were imported in the RT treatment planning system RayStation 11B DTK research version (RaySearch Laboratories, Stockholm, Sweden). For images without liver contours already available in the TPS, deep learning based segmentation was performed using a nnU-Net (Isensee *et al* 2021) trained on over 3000 CT scans collected at our institution. This process was used in this study only to speed up the generation of liver contours consistently across the longitudinal images, as all contours were visually reviewed and manually edited in the TPS when necessary. Rigid registration of the pre- and post-RT CT scans onto the planning CT was then performed in the TPS using the liver contours only. For each patient, all three CTs with their liver contours, the rigid registrations and dose distributions were exported from the TPS for processing and analysis.

To minimize a potential influence of the fractionation scheme differences among patients, all doses were converted to equivalent dose in 2 Gy fractions (EQD2):

$\mathrm{EQD}2=D\left(1+\frac{D}{n* \frac{\alpha }{\beta }}\right),$ with *D* the total dose, *n* the number of fractions and a standard coefficient *α*/*β* = 3 for the liver.

### Jacobian maps generation

DIR between the pre- and post-RT livers was performed using a biomechanical model driven by boundary conditions on the liver surface and vasculature centerline, as previously proposed (Cazoulat *et al* 2019). Briefly, the rigid registrations exported from the TPS were applied to the binary masks of the pre- and post-RT to align all images on the planning image and dose distribution. The registered binary mask of the pre-RT image was first converted into a triangular mesh and then into a solid tetrahedral finite-element model (FEM) with liver-specific elastic properties. DIR of the liver and auto-segmented vasculature masks were performed separately using a Demons algorithm. The results of these DIRs were used to determine boundary conditions to apply to the FEM of the pre-RT liver in a finite-element analysis (FEA) using the software Optistruct (Altair Engineering, Troy, Michigan). Displacements computed in each node of the FEM were then resampled into a dense displacement vector field (DVF) on the grid of the planning CT. Finally, the determinant of the Jacobian, noted |*J*|, was computed in each voxel of the DVF. Deformations outside the liver were discarded by considering no change in volume (|*J*| = 1). For a more convenient analysis, a negative offset of 1 was applied to these Jacobian maps so that negative and positive values respectively indicate a local contraction or an expansion in percent of the liver. In the rest of the study, Jacobian maps therefore designate (|*J*| −1).

### Analysis of the correlation between Jacobian and dose

**Figure 1.**
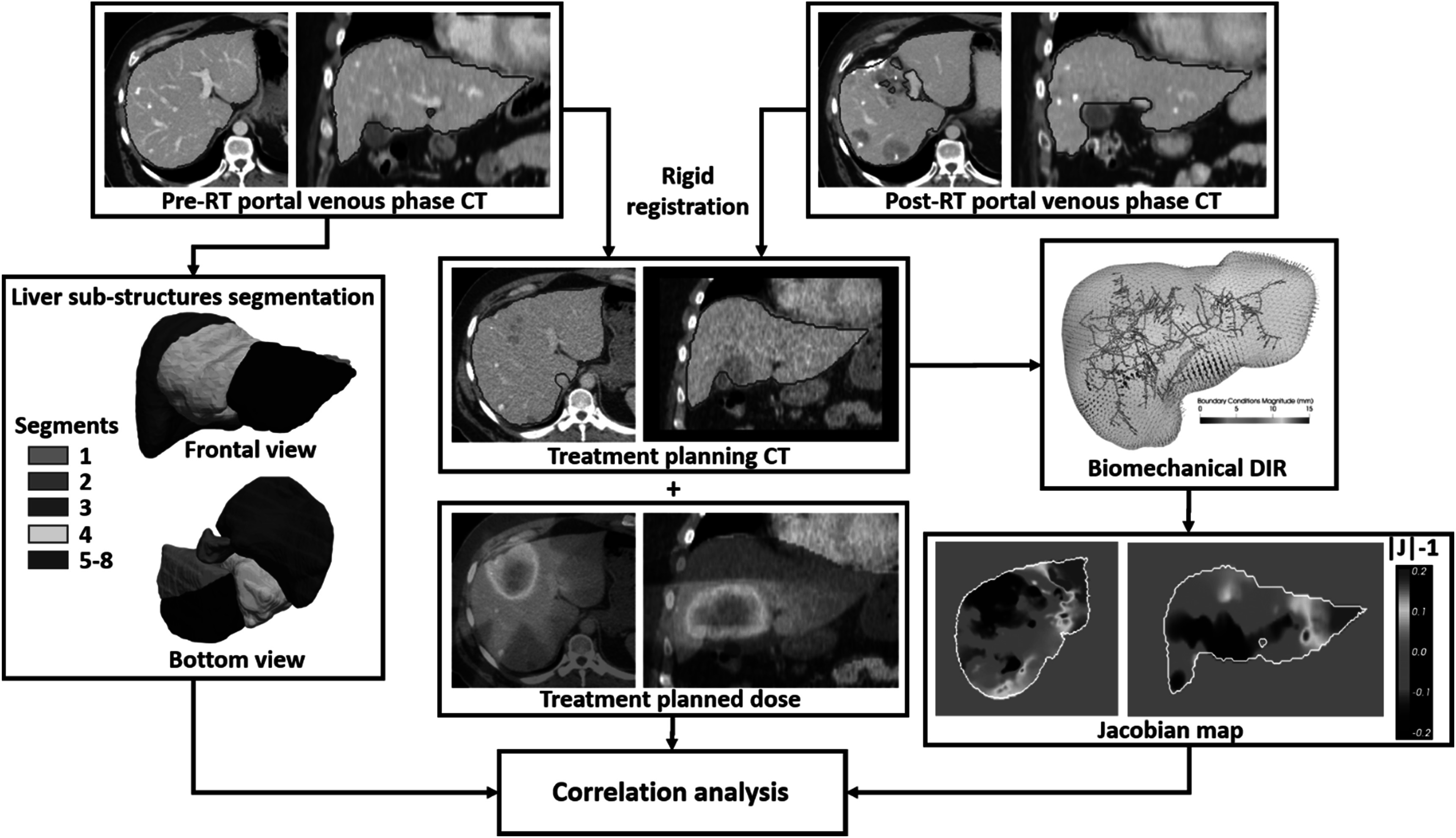
Workflow for the analysis of the relationship between planned dose and Jacobian maps.

Figure 1 represents the workflow for the analysis of the correlation between Jacobian maps and dose distribution in the whole liver and individual segments. The pre-RT and planning CTs are assumed to present with no or negligible deformations and only a rigid transformation was used to map the pre-RT image, on which are defined the sub-structures, and the dose distribution. After visual inspection of the fusion between rigidly mapped pre-RT CT and planned dose, the approximation associated with this rigid mapping of the dose was considered reasonable and preferred to a deformable approach which would introduce its own source of uncertainties in the proposed workflow. To define the segments in the pre-RT CT scan, a 3D full resolution nnN-Unet (Isensee *et al* 2021) previously trained in our group was used. The model was trained on 160 contrast-enhanced CTs collected at our institution under an IRB-approved protocol and carefully delineated by a radiologist (MA). As segments 5–8 were particularly challenging to delineate individually, they were combined in a single region defining the right lobe of the liver. The combination of segments 2–4 defined the left lobe. After segmentation of these sub-structures in the liver and biomechanical DIR between the pre- and post-RT livers, the dose and calculated Jacobian were averaged in the whole liver and each individual segment. The correlation between mean Jacobian, representing the volume change, and mean dose was measured using the Pearson coefficient *r*.

### Deep learning for Jacobian maps prediction

**Figure 2.**
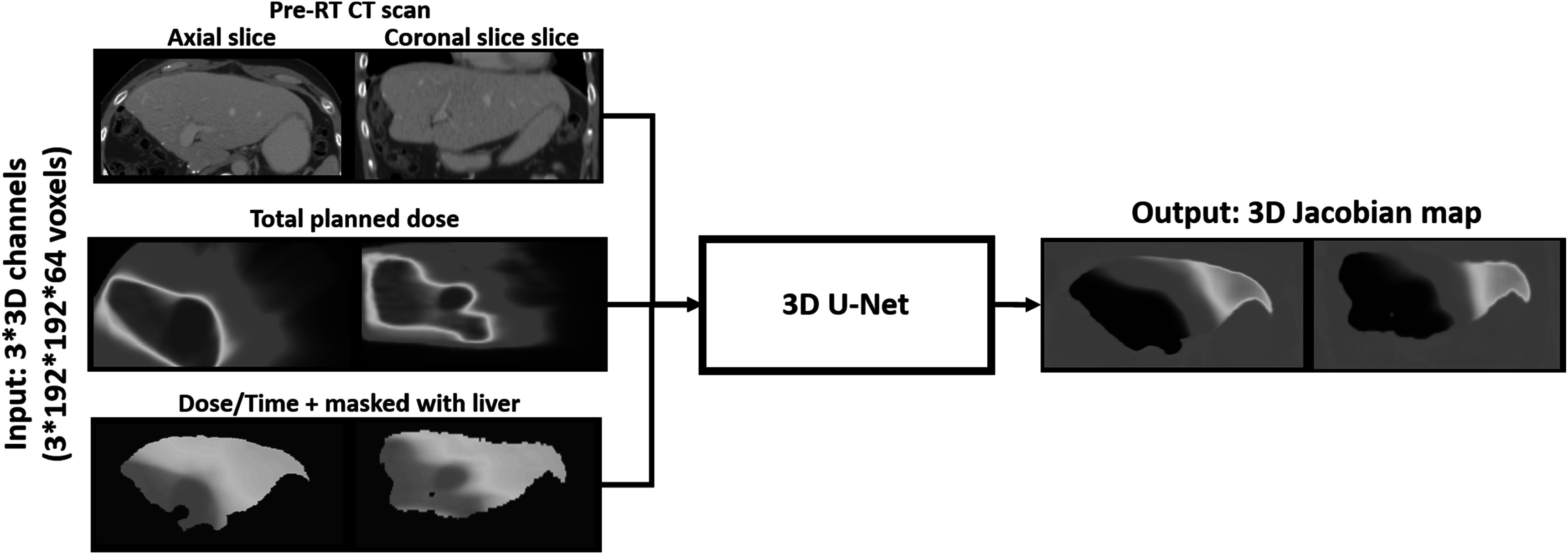
Representation of the inputs and output of the proposed U-Net model to predict liver radiation-induced volume change.

The use of a 3D U-Net architecture was evaluated to predict radiation-induced volume change of the liver solely based on the following pre-treatment data: diagnostic pre-RT CT, liver contour, planned dose distribution and number of days between end of RT and follow-up CT. This information was encoded in 3 channels as illustrated in figure 2. The first channel corresponded to the pre-RT CT with Hounsfield Units (HU) linearly scaled so that the range −1000 to 500 HU falls between 0 and 1, a standard practice to maximize the performance of the U-Net. The second channel corresponded to the planned EQD2 image with intensities divided by 100 to roughly fall in the same order of units. The third channel encoded both the information of the liver contour by setting all outside voxels to −1 and information of time gap *T* between end of RT and follow-up CT by setting all inside voxels to: EQD2/(T*100). To focus on the liver region, a grid of 192*192*64 voxels was automatically defined by the liver bounds and considering margins of 15 voxels on the left–right and anterior–posterior axes and 5 voxels on the superior-inferior axis. All 3 channels were resampled on this grid. Since liver varied in sizes this resampling process resulted in images with mean (min-max) voxel spacing varying between 1.32 (0.91–2.03), 1.15 (0.74–1.47) and 3.15 (1.90–4.42) mm in the left–right, anterior–posterior and inferior–posterior directions.

The 3D U-Net model employed in this study was the *BasicUnet* architecture implemented in the MONAI framework (Falk *et al* 2019, Cardoso *et al* 2022) with a dropout rate empirically set at 10%. The training aimed to minimize the mean squared error (MSE) between ground truth and predicted Jacobian maps using an Adam optimizer with a learning rate of 2.10^–4^.

Due to the limited size of the data, testing of the model was performed following a leave-10-out strategy. The dataset was split into 10 datasets, each containing 90 cases for training and 10 for testing. This split was stratified to ensure an equal repartition of the cancer types (HCC, CC or CR) between the 10 datasets. For each dataset, the MSE was optimized using 72 cases in batches of 2 and validated using 18 cases, after ensuring again equal proportions of cancer types between training and validation datasets. To augment the data, random rotations of −20° to +20° were applied around the three main axes. To reduce the influence of potential atypical cases on the validation metric, the MSE was calculated at the end of each epoch for the 18 evaluation cases but the retained validation metric was the average of the 16 smallest MSE. At each epoch, the model was saved if improving the previous best average MSE. The training was left running for 1000 epochs, a number ensuring no further improvement of the validation MSE.

### Analysis of the correlation between ground truth and predicted Jacobian maps

A voxel-wise comparison of the DIR-based and deep-learning predicted Jacobian maps was performed by measuring the Pearson correlation coefficient *r* for each of the 100 patients. The ability of the deep-learning model to establish more complex relationships between planned dose and Jacobian than a simple linear scaling of the dose was verified by comparing the distribution of coefficients *r* between deep-learning predicted and DIR-based Jacobian to the distribution of *r* between dose and DIR-based Jacobian. The performance of the model in estimating volume changes in all liver sub-regions was evaluated with the coefficient of determination between average deep-learning predicted and average DIR-based Jacobian. Finally, to evaluate the ability of the model in predicting hypertrophy or atrophy in each region, ROC areas under the curve (AUC) based on the predicted mean Jacobian were calculated.

## Results

### Mean EQD2 and Jacobian correlation

**Figure 3.**
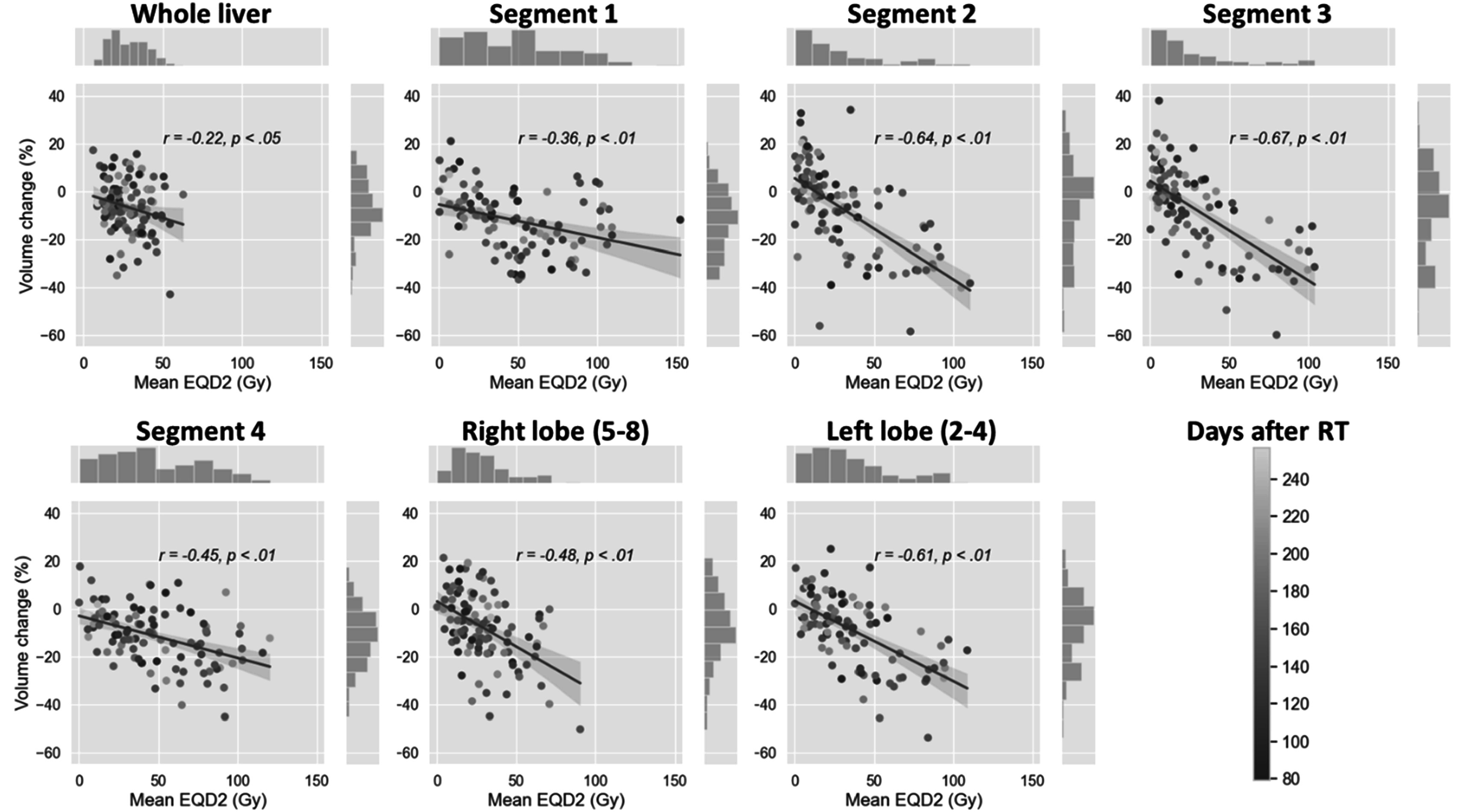
Scatter plots of the relationships between EQD2 and volume change estimated by the mean Jacobian for the whole liver, lobes and segments. The color of each data point represents the number of days between end of RT and follow-up.

Figure 3 represents the correlation between mean EQD2 and mean Jacobian for the whole liver, lobes and individual segments. Negative correlations were observed in all regions with Pearson *r* ranging from −22 for the whole liver to −0.67 for segment 3. Most hypertrophy cases (volume change > 0%) occurred for mean EQD2 < 50 Gy. No obvious effect of the time between end of RT and follow-up was observed. Table  2 summarizes the percentages of cases presenting with hypertrophy or atrophy for regions receiving EQD2 less and more than 50 Gy. Except when considering the whole liver, at least 90% of the regions receiving 50 Gy or more experienced volume decrease (and at least 72% a volume decrease greater than 10%). Among regions receiving less than 50 Gy, the proportion experiencing volume increase ranged from 18.6% for segment 4%–44.7% for segment 2. This volume increase was >10% only for 5% of segments 1 and 4. The linear regressions suggested that segments 5–8, 2 and 3 were the most sensitive to mean EQD2 with slopes of −0.38, −0.42 and −0.42, respectively. Segments 1 and 4 appeared the least sensitive to mean EQD2 with slopes of −0.14 and −0.18, respectively.

**Table 2.** Distribution of volume changes of the whole liver, lobes and segments based on EQD2.

	Percentage of cases					
	EQD2 < 50 Gy		EQD2 > 50 Gy		Linear regression Δ*V* = x*EQD2 + *y*	
	Δ*V* > 0%	Δ*V* > 10%	Δ*V* < 0%	Δ*V* < 10%	*X*	*Y*
Whole liver	28.4	7.5	60	40	−0.21	−0.77
Segment 1	22	5.1	90.2	78	−0.14	−5.53
Segment 2	44.7	21.1	91.3	82.6	−0.42	5.48
Segment 3	43	15.2	100	90	−0.42	4.4
Segment 4	18.6	5.1	90	72.5	−0.18	−2.92
Right lobe	33.7	11.6	100	71.4	−0.38	3.1
Left lobe	36.8	9.2	95.8	88.3	−0.34	3.25

**Figure 4.**
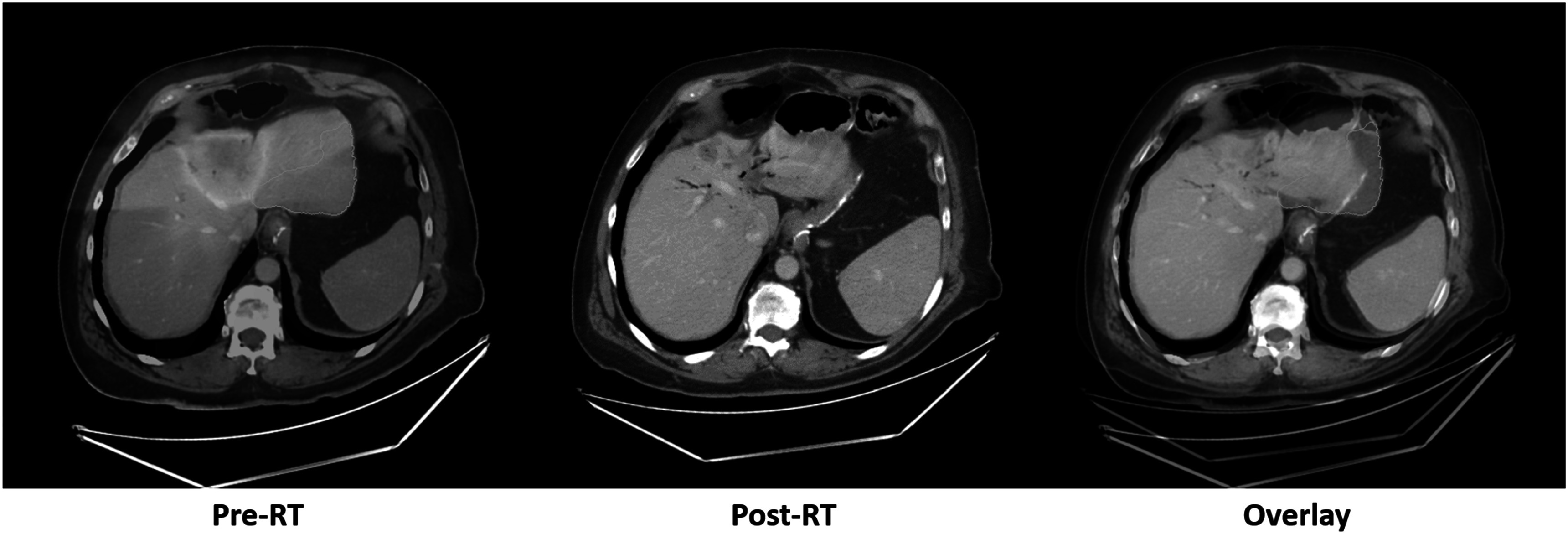
Example of an atypical case. Left: pre-RT CT scan with fused dose distribution and representation of segment 2 contour (cyan). Middle: post-RT CT scans exhibiting a clear shrinkage of the liver in the segment 2 region. Right: color overlay between the pre- and post-RT CT scans and representation of segment 2 (cyan).

Figure 4 illustrates an atypical case in the sense that the mean EQD2 in segment 2 was relatively low (15.8 Gy) but the corresponding volume decrease was one of the largest in the population (−56%). In this case, the mean EQD2 was the highest in segment 4 (91.4 Gy), suggesting that volume change of individual segments may also depend on the dose in neighboring segments.

### Correlation between ground truth and predicted Jacobian maps

**Figure 5.**
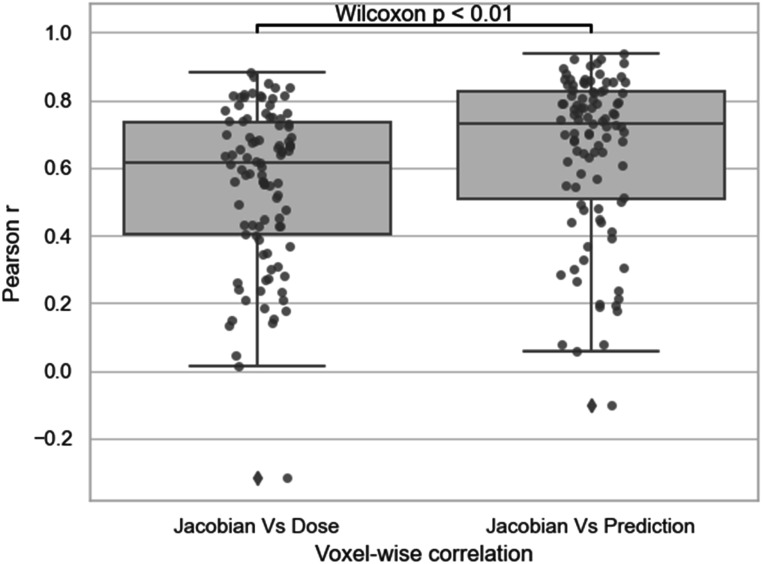
Distributions of the voxel-wise Pearson correlation r for the 100 patients between mean EQD2 and ground truth Jacobian (Left) and between predicted Jacobian and ground truth Jacobian (Right).

Figure 5 represents the distributions of the Pearson correlations *r* at the voxel level between deep-learning predicted and DIR-based Jacobian maps and the opposites of *r* between EQD2 and DIR-based Jacobian maps. As the two distributions deviated significantly from normality according to a Shapiro test (*W* = 0.88, *p* < 0.01 and *W* = 0.93, *p* < 0.01, respectively), the statistical significance between the two means was assessed with a Wilcoxon signed ranked test. The mean correlation between DIR-based and deepr-learning predicted Jacobian maps was significantly higher than the mean correlation between dose and ground truth Jacobian (0.63 ± 0.24 versus 0.55 ± 23, *p* < 0.01).

**Figure 6.**
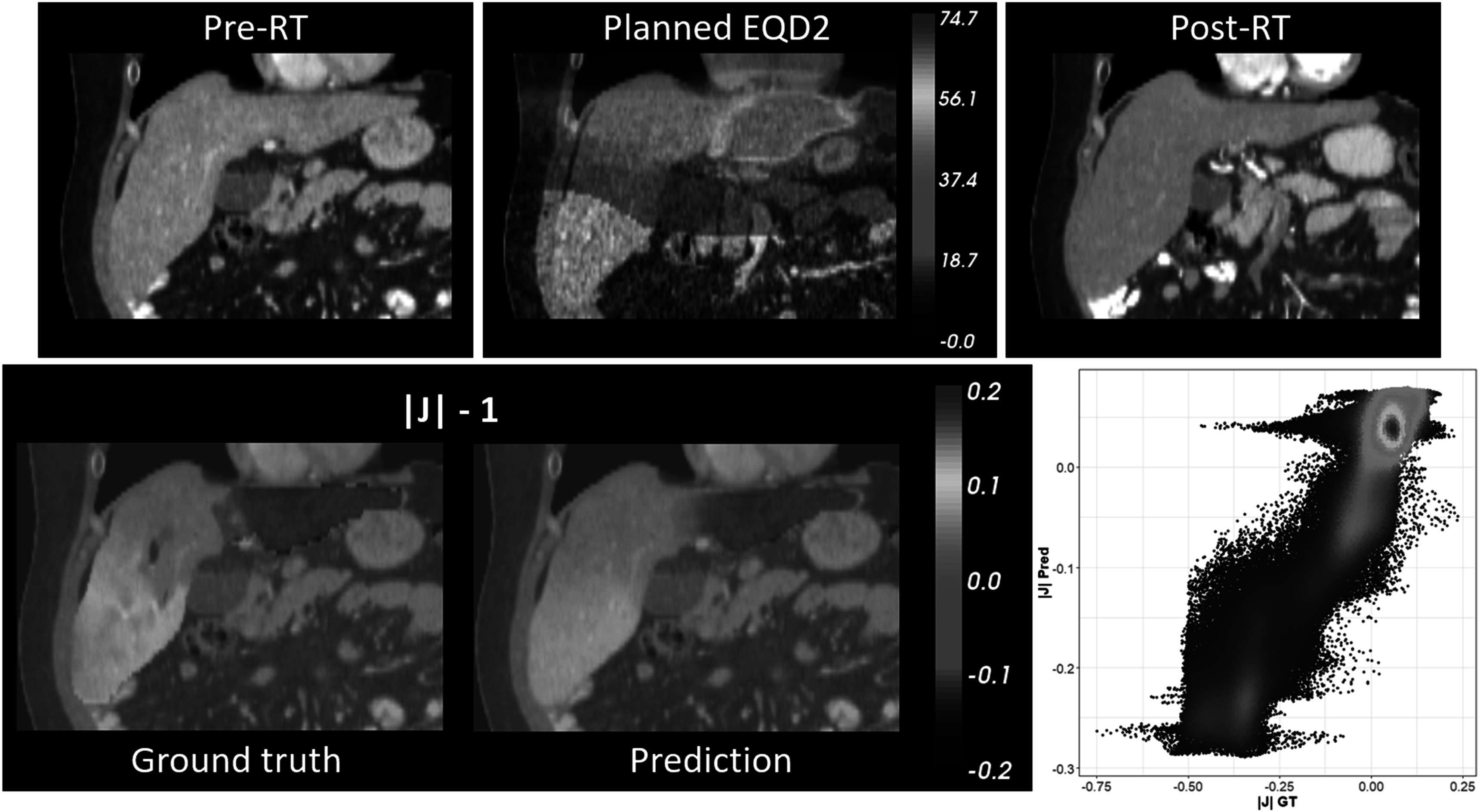
Example of good a prediction result (*r* = 0.93); prescription: 67.5 Gy in 15 fractions; time between end of RT and followup: 172 d; DIR-based versus deep-learning predicted volume change: left lobe −28.7%/−17.7%, right lobe +3.8%/+2.7% correlation result between deep-learning predicted and DIR-based Jacobian.

Figure 6 illustrates an example of a case with high correlation at the voxel level between deep-learning predicted and DIR-based Jacobian (*r* = 0.93). In this case, the high dose was prescribed to the left lobe which, according to the DIR-based Jacobian, led to a clear shrinkage of this lobe (|J| −1 < 0) and expansion of the right lobe ((|J| −1 > 0). By averaging the Jacobian values in the lobes, this corresponded to a shrinkage of 28.7% or 17.7% according to the DIR-based or deep-learning prediction, respectively. The right lobe, which received much less dose, experienced an expansion of 3.8% or 2.4% according to the DIR-based or deep-learning prediction, respectively.

**Figure 7.**
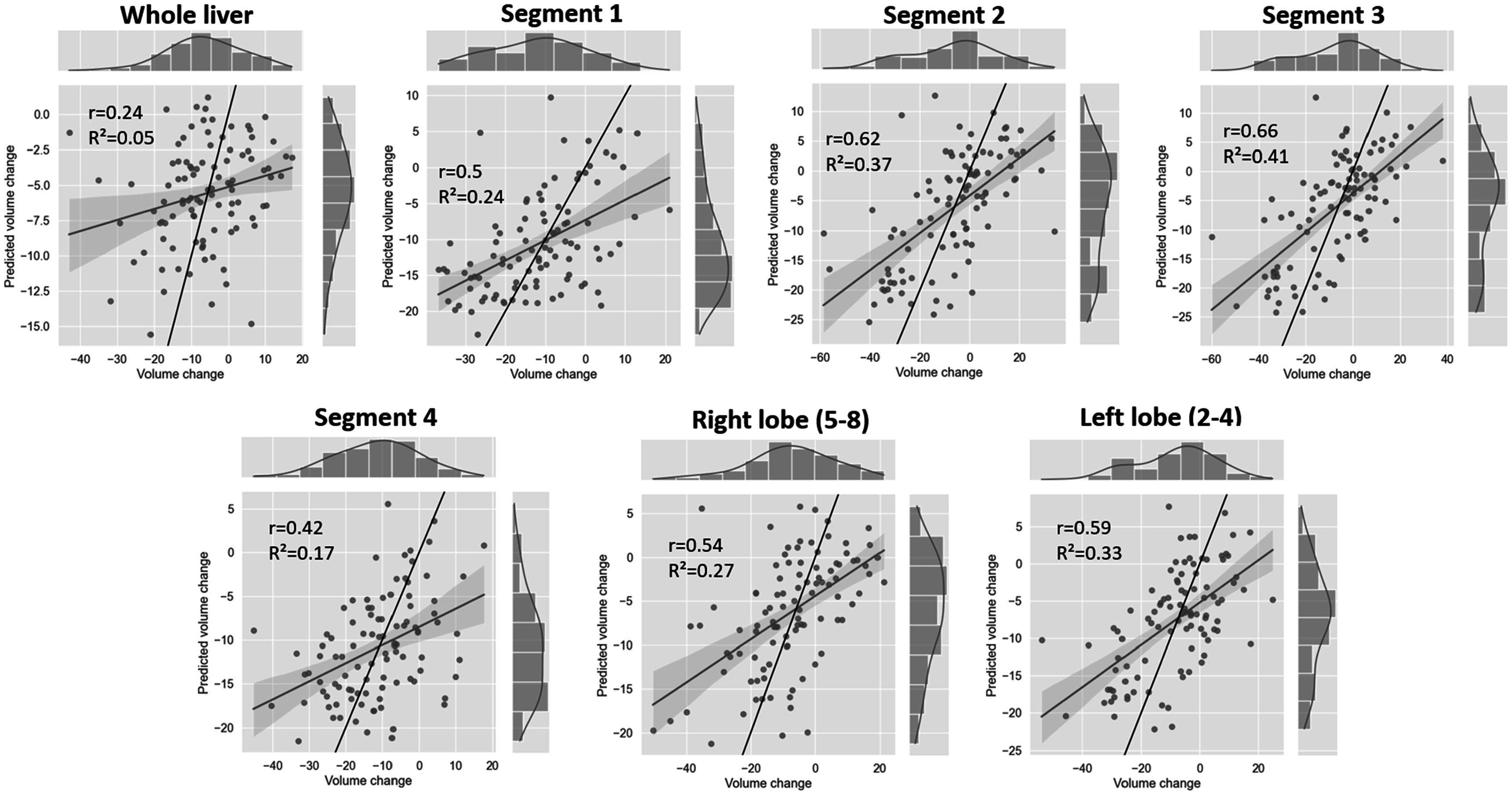
Representation of the correlation between deep-learning predicted and DIR-based volume changes in different liver regions with Pearson r and coefficients of determination *R* ^2^.

Figure 7 compares the deep-learning predicted and DIR-based volume changes calculated by averaging the Jacobian in the whole liver and different sub-regions. The correlation coefficients *r* ranged from 0.24 for the whole liver to 0.66 for segment 3, which was similar to the coefficients reported between planned EQD2 and DIR-based Jacobian, with the exception of segment 1 and the right lobe for which the proposed deep-learning prediction model led to some improvement of the correlation (from 0.24 to 0.5 and from 0.48 to 0.54, respectively). The coefficients of determinations were all relatively low, with a maximum for segment 3 (*R* ^2^ = 0.41), as the deep-learning prediction model generally underestimated the magnitude of the volume change and estimated too few cases of actual hypertrophy.

**Figure 8.**
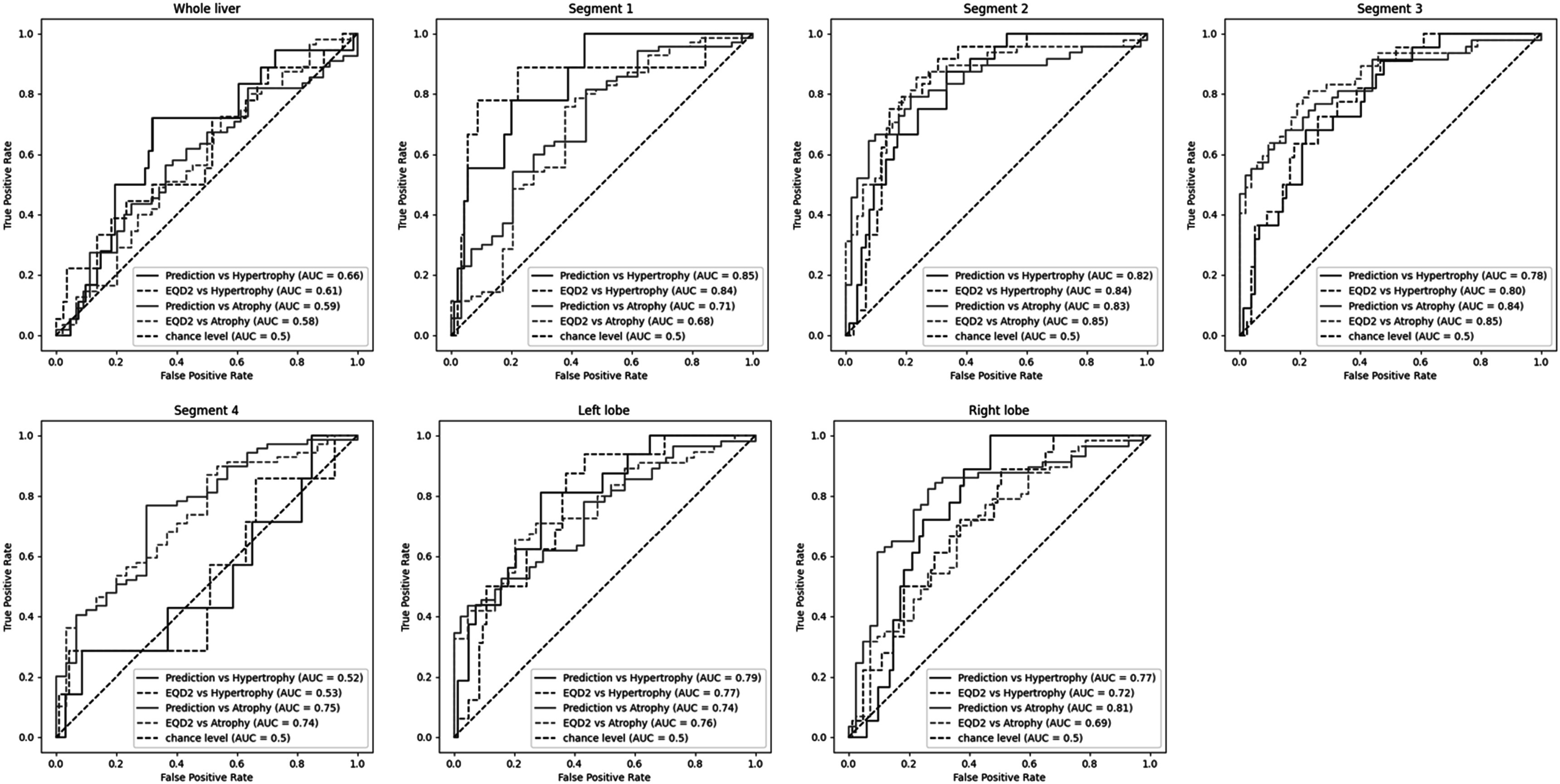
ROC curves for the following definitions: hypertrophy: Δ*V* > 5%; atrophy: Δ*V* < 5%.

To compare the performance of the deep-learning prediction model to the sole information of mean planned EQD2 in classifying cases of atrophy (defined as Δ*V* < 5%) and hypertrophy (defined as Δ*V* > 5%), ROC AUC were calculated and reported in figure 8. Both the deep-learning predicted Jacobian and planned mean EQD2 led to similar and fair AUC results, except for the prediction of hypertrophy cases of segment 4. Only for the largest considered volumes, the whole livers and right lobes, the deep-learning predicted Jacobian performed noticeably better than the mean dose with AUC of 0.66 versus 0.61 and 0.77 versus 0.72 for the hypertrophy cases, 0.59 versus 0.58 and 0.81 versus 0.69 for the atrophy cases.

## Discussion

This study proposed the use of DIR to generate volume change maps in the liver months after radiation therapy. Using these maps, a clear effect of the planned dose distribution on the volume change was observed in different regions of the liver defined by single or combinations of Couinaud segments. While mean EQD2 in the whole liver appeared as a poor predictor of global liver volume change (*r* = −022), moderate correlations between mean EQD2 and volume change was observed for all liver sub-regions analyzed individually (*r* range: −0.36; −067). Most cases of estimated hypertrophy occurred in regions receiving lower dose (EQD2 < 50 Gy) but hypertrophy was a relatively rare outcome in this cohort of patients with only about 10% of the left and right lobes showing a volume increase above 10%. Optimization of the dose distribution during treatment planning to maximize the chances of hypertrophy may help in preserving the overall liver function. However, mean dose alone was a poor predictor of hypertrophy for all liver segments. In the example illustrated in figure 4, the shrinkage of segment 2 could be due to the high dose received in the neighboring segment 4 which potentially compromised the vascularization of other parts of the liver. In addition to such complex spatial relationships, volume changes are expected to depend on many patient-specific characteristics.

We hypothesized that the diagnostic pre-RT CT scan contains valuable patient-specific information for the volume change prediction model, such as the size, texture and shape of the liver and associated disease. The use of a deep learning U-Net model was evaluated to automatically extract features from the Pre-RT CT scan and dose distribution and to reconstruct a prediction of the volume change map. The predicted volume change maps showed a significantly stronger voxel-wise correlation with the DIR-based volume change maps than when considering the original dose distribution, demonstrating the ability of the model to establish a more complex relationship between dose and volume change than a simple linear scale. However, the magnitude of the predicted volume change in each segment was generally underestimated by the model which predicted very few hypertrophy cases. A ROC AUC analysis showed that thresholding the mean predicted volume change or original mean EQD2 lead to similar performance to classify individual segments as experiencing hypertrophy or not (defined as a volume change greater than 5%). When considering larger volumes, for example, the left or right lobes or the whole liver, the dose was more likely heterogeneous and therefore its mean value less relevant. In this case, averaging the deep-learning predicted Jacobian in each voxel of the considered liver volume led to a stronger classification performance. Considering the small percentage of segments presenting with volume change greater than 10% in our cohort of 100 patient (between 5% and 21%), we believe that the deep-learning model performance can be improved by training the U-Net with a larger dataset when data become available. In particular, we expect that balancing techniques to include a larger proportion of hypertrophy cases will improve the performance in the prediction of such cases. To minimize the heterogeneity in treatment prescriptions, we included in this study only patients who received an IMRT prescription in the recent years of at least 50 Gy and in at least in at least 10 fractions. Doses were then transformed into equivalent dose in 2 Gy fractions (EQD2) to minimize the impact of the fractionation scheme differences. These criteria, in addition to the requirement of very specific imaging data, limited the number of patients we could identify to be included in this study. As we consider our current dataset size as already small to train a deep-learning model, no additional stratification based on prescription or cancer type was conducted. Ideally, the promising preliminary results reported in this paper will motivate the collection of similar data in other institutions which would also enable external validation of the model.

This study has some other limitations. First, the considered ground truth Jacobian was generated automatically using DIR. Even if the biomechanical DIR model has been previously optimized specifically for the registration of longitudinal liver CT scans (Cazoulat *et al* 2019), the resulting Jacobian maps could still be prone to some uncertainties. The significantly better target registration error achieved by this DIR method in comparison to others of reference (Toesca *et al* 2017), suggests that it also allows to provide the best estimate of volume change at the voxel level. However, evaluation of the accuracy of such maps is complex due to the limited number of anatomical details in the liver images. The use of an alternative more accurate DIR method, if available, may lead to stronger correlations between Jacobian and dose. An inconvenience of this DIR method is the requirement of accurate liver contours, but the continuous progress in deep learning-based segmentation models keeps facilitating the generation of these contours and reducing the need for manual correction. In this study, all liver contours were carefully reviewed and edited manually when necessary to maximize the DIR accuracy as in a previous study (Toesca *et al* 2017). The optimization of the liver segmentation tool and its evaluation were therefore out of the scope of this study but will be carried out separately which will facilitate the analysis of future data as they become available. Similarly, accuracy of the employed segmentation algorithm for the definition of liver segments remains to be validated. However, this tool was used only to generate a rough splitting of the liver on the pre-RT CT scan and, due to the smoothness of the spatial distributions of both the dose and Jacobian images, we expect small variations in this splitting to have a minor impact on the estimated relationship between mean dose and mean Jacobian. Future investigations will compare the volume changes estimated with DIR to the volume changes measured after auto-segmentation of both the pre- and post-RT images. Using this second method, it is still unclear what consistency can be achieved in the definition of liver segments between the longitudinal images, even in case of manual editing by an expert. Moreover, an advantage of the DIR approach proposed in this paper is the estimation of the volume change at the voxel level. In the same fashion as using voxel-based pre-treatment liver perfusion maps (Wu *et al* 2016), such Jacobian maps could be integrated in the dose optimization algorithm instead of using constraints on macroscopic sub-regions like liver segments. Another use of the Jacobian maps could be to simulate dose-induced volume change in finite-element analysis as proposed by Polan *et al* (2017) who demonstrated improvement in DIR accuracy by initializing the deformation with expected radiation-induced volume variations. This improvement in DIR accuracy should allow more accurate dose mapping between past and current planning CT scan in case of re-irradiation of the liver, especially when contrast is limited or inconsistent between the two treatment planning CT scans. In conclusion, we believe the automatic generation of predicted volume change maps described in this paper represents a very promising tool for the development of future adaptive and personalized liver RT strategies.

## Data Availability

The data cannot be made publicly available upon publication because no suitable repository exists for hosting data in this field of study. The data that support the findings of this study are available upon reasonable request from the authors.

## Ethical statement

This retrospective study was approved by The University of Texas MD Anderson Cancer Center Institutional Review Board (PA18-0832). The requirement for written informed consent was waived for this retrospective analysis.
